# Antioxidant Activity and Phenolic Content of Microwave-Assisted* Solanum melongena* Extracts

**DOI:** 10.1155/2014/719486

**Published:** 2014-02-09

**Authors:** Loredana Salerno, Maria N. Modica, Valeria Pittalà, Giuseppe Romeo, Maria A. Siracusa, Claudia Di Giacomo, Valeria Sorrenti, Rosaria Acquaviva

**Affiliations:** Dipartimento di Scienze del Farmaco, Università degli Studi di Catania, Viale A. Doria 6, 95125 Catania, Italy

## Abstract

Eggplant fruit is a very rich source of polyphenol compounds endowed with antioxidant properties. The aim of this study was to extract polyphenols from eggplant entire fruit, pulp, or skin, both fresh and dry, and compare results between conventional extraction and microwave-assisted extraction (MAE). The effects of time exposure (15, 30, 60, and 90 min) and solvent (water 100% or ethanol/water 50%) were also evaluated. The highest amount of polyphenols was found in the extract obtained from dry peeled skin treated with 50% aqueous ethanol, irradiated with microwave; this extract contained also high quantity of flavonoids and showed good antioxidant activity expressed by its capacity to scavenge superoxide anion and to inhibit lipid peroxidation.

## 1. Introduction

Polyphenols have received a great deal of attention in recent years due to their powerful antioxidant properties. Polyphenols are present at high concentrations in a variety of fruits and vegetables. Many studies have demonstrated that consumption of fruits and vegetables rich of polyphenols is related to a reduced risk of coronary heart diseases, neurodegenerative diseases, and certain forms of cancer [1–3].

With the aim of studying vegetables with antioxidant properties, we focused our interest on eggplant (*Solanum melongena* L.). Eggplant is a worldwide diffused vegetable and a common food; the whole eggplant fruit possesses antioxidant activity and is ranked among the top ten vegetables in terms of oxygen radical absorbance capacity [4]. Antioxidant activity of eggplant was tested by different assays [5]. There are several research publications describing phenolic compounds extracted from eggplant and the health benefits associated with their use. Besides antioxidant activity of entire fruit [6], pulp [7], or skin [8, 9], a number of pharmacological properties were reported: hepatoprotective [10], anti-inflammatory [11], hypolipidemic [12], antiallergic [13], and anticancer [14]. Usually, observed activities well correlated with the total phenolic content of the eggplant fruit.

The quantity and quality of phenols present in fruit and vegetables may be significantly influenced by cultivar, environment, soil type, growing, storage conditions, and cooking. Luthria et al. [15] did not observe significant difference in the total phenolic content of eggplant samples grown with organic and conventional farming practices. Scalzo et al. [16] studied changes in antioxidant activity of aqueous eggplant extracts before or after grilling and boiling, concluding that thermal treatment commonly used before consumption of this vegetable can increase the content and biological activity of antioxidant compounds present in eggplants. Moreover, quantity and quality of phenols may depend on extraction procedures. Usually traditional methods, such as solvent extraction, were employed to study the phenolic composition of eggplant [17, 18].

In the recent years, faster and more efficient novel techniques including ultrasound-assisted extraction, microwave-assisted extraction (MAE), supercritical fluid extraction, and accelerated solvent extraction have been developed for the extraction of nutraceuticals from plants and foods [19]. These techniques often shorten the extraction time, decrease the solvent consumption, increase the extraction yields, and enhance the quality of extracts because of reduced risk of degradation of thermolabile constituents. A number of recent reports suggested that extraction of phenolic compounds from various sources and antioxidant capacity could be enhanced via MAE [20–23]. To the best of our knowledge there are no reports on MAE of phenolic compounds from eggplant; the present study was designed to investigate the utility of MAE to extract phenolic antioxidant compounds from eggplant entire fruit, pulp, or skin, both fresh and dry; a comparison was made between conventional extraction and MAE. The effects of time exposure (15, 30, 60, and 90 min) and solvent (water 100% or ethanol/water 50%) were also evaluated. Water and ethanol were chosen as solvents for environmental reasons and possible food application. The extract containing the higher quantity of polyphenols was further analyzed to determine the flavonoid content and antioxidant activity. To this purpose, the ability of this extract to scavenge superoxide anion, to quench a stable radical, and to inhibit *in vitro* lipid peroxidation was evaluated.

## 2. Materials and Methods

### 2.1. Materials

Eggplant was purchased from a local distributor. Gallic acid, Folin-Ciocalteu reagent, and nicotinamide-adenine dinucleotide reduced form (NADH), catechin, 1-diphenyl-2-picrylhydrazyl (DPPH), and xylenol orange were purchased from Sigma-Aldrich Co. (St. Louis, MO, USA). All reagents and solvents used were of reagent grade unless otherwise specified.

### 2.2. Sample Preparation and Extraction Procedures

The fruit was cut into pieces and homogenized (entire eggplant). The peeled skin and pulp were homogenized separately. All the fresh samples were kept at −20°C until use. A number of homogenates were lyophilized to obtain dry samples, which were kept at −20°C until use.

Polyphenolic compounds were extracted from eggplant entire fruit, skin, or pulp (fresh or dry) using a conventional method or a microwave instrument (Focused Microwave Synthesis System CEM Discovery). The extraction variables evaluated were irradiation time (15, 30, 60, and 90 min) and solvent (deionized water 100% and 50% aqueous ethanol). A constant temperature (100°C) and a constant solvent volume of 50 mL for 1.5 g of fresh or dry entire eggplant, pulp, or skin were chosen for the current study. Dry entire eggplant, pulp and skin were obtained by lyophilization from 24, 25, and 20 g of fresh samples, respectively.

#### 2.2.1. Microwave-Assisted Extraction

Microwave irradiation was accomplished with a CEM Discovery instrument and took place in 80 mL closed reactor vessels containing accurately weighed samples. A stir bar was placed in each reactor vessel, which was then inserted into the microwave oven. Conditions within the reaction vessel were power 150 W, pressure 150 Psi, temperature 100°C, run time 2 min, and then the time selected for each experiment (15, 30, 60, and 90 min). After microwave treatment, samples were allowed to cool and then an amount of mixture was transferred to a test tube for centrifugation; the supernatant was used for the total phenols determination.

#### 2.2.2. Conventional Extraction

Fresh or dry entire eggplant, pulp, or skin were suspended in solvent, agitated with stir bar, and then heated in an oil bath with a temperature control system (Heidolph EKT 3001) performed at 100°C for the chosen time. Following treatment, the mixture was cooled and then an amount of mixture was transferred to a test tube for centrifugation; the supernatant was used for determination of total phenols.

### 2.3. Total Phenols Determination

The concentration of total phenolic content was determined spectrophotometrically, using the Folin-Ciocalteu total phenols procedure, described by Ballard et al. [20], with slight modifications. Gallic acid standard solutions were prepared at 0.0, 0.0125, 0.025, 0.050, 0.100, and 0.150 mg/mL. Test extracts (0.1 mL) appropriately diluted or the gallic acid standards (0.1 mL) were transferred to 15 mL test tubes. Folin-Ciocalteu reagent (0.2 N, 3.0 mL) was added to each test tube and mixed using a vortex mixer. After 1 min, 2.0 mL of 9.0% (w/v) Na_2_CO_3_ in water was added and the obtained solution was mixed and left at room temperature. After 2 h, absorbance was determined at *λ* = 765 nm, using a PriXma UV 1200 spectrophotometer. The concentration of total phenolic compounds in the extracts was determined comparing the absorbance of the extract samples to that of the gallic acid standard solutions. All samples were analyzed in triplicate. Total phenolic content (TPC) was expressed as gallic acid equivalents (GAE) in mg per 1.5 g fresh or dry eggplant.

### 2.4. Total Flavonoid Content

The total flavonoid concentration was measured using a colorimetric assay described by Zhishen et al. [24], with slight modifications. Catechin standard solutions were prepared at 0.0, 0.004, 0.008, 0.016, 0.032, and 0.064 mg/mL. Briefly, 25 *μ*L of the eggplant extract and/or catechin standard solutions was added to a 100 *μ*L of water. At time zero, 7.5 *μ*L of 5% NaNO_2_ was added; at 5 min, 7.5 *μ*L of 10% AlCl_3_ was added; at 6 min, 50 *μ*L of 1 M NaOH was added. Each reaction mixture was then immediately diluted with 60 *μ*L of water and mixed. Absorbances of the mixtures upon the development of pink color were determined at *λ* = 510 nm relative to a prepared blank. The total flavonoid content of the samples is expressed as catechin equivalents (CE) in mg per 1.5 g extract. All samples were determined in triplicate.

### 2.5. Antioxidant Activities

Based on results regarding polyphenolic and flavonoid content, the extract obtained from dry peeled skin treated with 50% aqueous ethanol solution under microwave-assisted extraction (EPS50MAE) was selected to evaluate antioxidant activities. EPS50MAE was dried on Rotavapor and the dry mass was dissolved in phosphate buffered saline (PBS).

#### 2.5.1. Quenching of DPPH

The free radical scavenging capacity of eggplant extract was tested by its ability to bleach the stable DPPH radical. The reaction mixture contained 86 *μ*M DPPH and different amounts of EPS50MAE in a total volume of 1 mL of ethanol to obtain concentrations of EPS50MAE ranging from 8.75 to 875 *μ*g/mL. After 10 min at room temperature the absorbance at *λ* = 517 nm was recorded. Results are expressed as percentage decrease in absorbance with respect to control. Trolox (30 *μ*M), a water-soluble derivative of vitamin E, was used as standard; it had a quenching activity of 100%.

#### 2.5.2. Scavenger Effect on Superoxide Anion

Superoxide anion was generated *in vitro* as reported by Acquaviva et al. [25]. The assay mixture contained in a total volume of 1 mL : 100 mM triethanolamine-diethanolamine buffer, pH 7.4, 3 mM NADH, 25 mM/12.5 mM ethylene diamine tetra-acetic acid (EDTA)/MnCl_2_, and 10 mM *β*-mercaptoethanol; some samples contained EPS50MAE at different concentrations of (875–8.75 *μ*g/mL). After 20 min incubation at 25°C, the decrease in absorbance was measured at *λ* = 340 nm. Results are expressed as percentage of inhibition of NADH oxidation with respect to control. Superoxide dismutase (SOD) 80 mU/mL was used as standard; it had a scavenging activity of 100%.

#### 2.5.3. Determination of Lipid Hydroperoxide Levels in the Plasma of a Healthy Donor

Plasmatic lipid hydroperoxide levels were evaluated by oxidation of Fe^2+^ to Fe^3+^ in the presence of xylenol orange (FOX assay) at *λ* = 560 nm [26]. Heparinized venous blood was collected after overnight fasting; plasma was separated by centrifugation at 800 g for 20 min. Plasma aliquots (500 *μ*L) were diluted 1 : 1 with oxygenated PBS and incubated at 37°C for 2 h with or without different concentrations of EPS50MAE (875–8.75 *μ*g/mL) in a total volume of 1 mL. Calibration was obtained using hydrogen peroxide (0.2–20 *μ*M). Results are expressed as percentage of inhibition with respect to control (plasma incubated in absence of test compounds).

#### 2.5.4. Statistical Analyses

The data are presented as means ± SD for 4 experiments in triplicate. One-way variance analysis and Student's *t*-test were used where appropriate; *P* < 0.05 was regarded as significant.

## 3. Results and Discussion

### 3.1. Polyphenolic and Flavonoid Content

It is known that numerous fruits and vegetables contain high amount of polyphenols which can be beneficial for human health [27]. Eggplant is listed among the top ten vegetables in terms of oxygen radical absorbance capacity [4]. In addition, this plant is consumed throughout the world and it is endowed with many potential pharmacological properties [7, 9–14]. As first aim, in this research we wanted to determine the amount of polyphenolic compounds in various parts of the vegetable (skin, pulp, or entire fruit) and to compare the effect of MAE with a conventional method. Results are summarized in Tables 1 and 2 and are expressed as mg gallic acid equivalents on 1.5 g of eggplant. Firstly we determined the content of polyphenolic compounds on samples of both fresh and dry entire fruit, pulp, and skin, using both water and 50% aqueous ethanol as solvents, at 100°C for a fixed time of 15 min (Table 1). Methanol or other organic extraction solvents, instead of ethanol, often used for the extraction of polyphenols from plants and food [10], were avoided for their toxicity that would not allow the use of these data for food application. Among the various parts of eggplant examined, peeled skin contains the major amount of polyphenolic compounds with respect to entire fruit and pulp, both in fresh and dry samples. These results suggest the use of skin, waste production of eggplants, as source of polyphenols for nutraceutical extraction; in addition, content of polyphenols in dry samples is greater than that in fresh, but not proportional to the amount of starting fresh materials used. This is probably due to the matrix of the dry samples that does not permit an extraction similar to that of the fresh. However, comparison of the results in fresh and dry eggplant suggests consumption of entire fresh fruit for beneficial salutary effects since the quantity of polyphenols present is sufficient to contribute to the daily intake of bioflavonoids [28]. The effects of various investigated parameters (microwave irradiation and solvents), were different if we compare fresh with dry materials. Usually, microwave irradiation with respect to conventional method and solvent composition (50% aqueous ethanol with respect to 100% water), significantly increase the polyphenol extraction for dry materials in all examined samples, entire fruit, pulp and skin. On the contrary, generally both parameters do not influence polyphenols extraction for fresh materials. In a next step we compared the content of polyphenolic compounds in samples of dry entire fruit or skin at different heating times (15, 30, 60 and 90 min) using 100% water. Results, showed in Table 2, demonstrate that heating time increases the yield of polyphenolic compounds and microwave irradiation positively affects this yield on entire plant, but not on skin. Finally, we tested the effect of the employment of the use of 50% aqueous ethanol solution to determine the quantity of polyphenols in dry entire fruits and skin maintained at 100 °C for 90 min, with conventional and microwave irradiation; water/ethanol showed to be very effective solvent particularly on skin treated with microwave irradiation. Consequently, the extract obtained from dry peeled skin treated with 50% aqueous ethanol solution under microwave-assisted extraction for 90 min (EPS50MAE) was selected for further analyses. In this extract the content of flavonoid compounds was also determined. Results demonstrate that EPS50MAE contained 13.5 mg CE/1.5 g extract which well correlated with the amount of total phenolic compounds.

### 3.2. Antioxidant Activities

Antioxidant activity of EPS50MAE in this study was assessed by the use of three tests: (i) *in vitro *quenching of DPPH radical, (ii) *in vitro *scavenger effect on superoxide anion, and (iii) “*ex vivo cell-free system*” to evaluate lipid peroxidation inhibitory activity.

Free radical species such as superoxide and hydrogen peroxide have both been implicated in several diseases such as atherosclerosis, chronic inflammation, and cancer. In particular, it is now recognized that the extremely reactive hydroxyl radical (^∙^OH) derived from O_2_^−^ and H_2_O_2_ causes DNA strand scission in cellular damage [29]. The importance of removing active oxygen species from living organisms is becoming clear, while there is growing interest in the protective mechanisms whereby antioxidants that scavenge reactive oxygen species (ROS) may have a potential therapeutic use [30].

The free radical scavenging activity of EPS50MAE was tested by its ability to bleach the stable DPPH radical [25]. This assay provides information on the reactivity of test sample with a stable free radical. Because of its odd electron, DPPH gives a strong absorption band at 517 nm in visible spectroscopy (deep violet color). As this electron becomes paired off in the presence of a free radical scavenger, the absorption vanishes, and the resulting decolorization is stoichiometric with respect to the number of electrons taken up. In this assay, this extract did not show a significant DPPH quenching capacity, probably due to the large size and high stability of this radical. In addition it was not possible to test higher concentrations because the reaction mixture became opalescent.

Since the antioxidant effects of polyphenols could be due both to their free radical scavenging and/or chelating activities, to investigate the superoxide anion scavenging capacity of the extract, we used a method which excludes the Fenton-type reaction and the xanthine/xanthine oxidase system. Results reported in Table 3 are expressed as IC_50_ (*μ*g/mL) which represents the concentration of EPS50MAE required to scavenge 50% superoxide anion formation. EPS50MAE inhibited superoxide anion formation in a dose-dependent manner with IC_50_ value of 17 ± 0.3 *μ*g/mL; this last result would suggest that components of the extract are able to scavenge the small free radical superoxide anion and then contribute to antioxidant activity of EPS50MAE.

There is growing evidence that oxidative stress may represent one of the agents involved in the initiation and/or progression of many human diseases including atherosclerosis, postischemic reperfusion injury, inflammation, aging, and neurodegenerative pathologies (Parkinson's and Alzheimer's diseases). In addition, several experimental results link the overproduction of ROS to biological damage. ROS, in fact, are able to chemically alter virtually all the major classes of biomolecules (proteins, nucleic acids, and lipids). Like other aerobic organisms, humans have evolved a variety of mechanisms to protect themselves from the potentially deleterious effects of ROS; nevertheless, oxidative stress occurs when the generation of free radicals exceeds the capacity of a cell to defend or repair itself.

Since* in vitro* studies have shown that phenolic compounds may prevent lipid peroxidation and formation of atherosclerotic plaques [31, 32], in this study the antioxidant activity of EPS50MAE was also evaluated by measuring antilipoperoxidative capacity in human plasma incubated with or without extract for 2 h at 37°C. Results of this study showed that the addition of EPS50MAE determined an inhibition of lipid peroxidation (Table 3) even though this effect was moderate with respect to superoxide anion scavenging activity. The ability of EPS50MAE to counteract *in vitro* plasmatic lipid peroxidation may contribute to prevention of the formation of atherosclerotic plaques and subsequent cardiovascular diseases.

## 4. Conclusions

Antioxidant activity of polyphenols may have health-promoting and disease-preventing effects. Consequently, consumption of eggplant, which contains polyphenols, or extracts of polyphenols from eggplant, may have a potential therapeutic use. Our study confirms that polyphenols are present in various parts of eggplant, in particular skin, suggesting the use of the entire vegetable as food; MAE may be an attractive alternative to conventional method to improve the amount of polyphenols extracted, in particular for nutraceutical application. Extraction of skin with 50% aqueous ethanol with microwave irradiation was particularly effective. In addition, our study provides evidence that EPS50MAE exhibits interesting antioxidant properties, expressed by its capacity to scavenge superoxide anion and to inhibit lipid peroxidation.

## Figures and Tables

**Table 1:** Phenolic content of water extracts and 50% aqueous ethanol extracts of fresh and dry entire eggplant, pulp, and skin at 100°C for 15 min, with conventional method or microwave-assisted extraction (MAE). Values are reported in mg gallic acid equivalents ± SD (*n* = 3) on 1.5 g of eggplant.

	Fresh (water)	Fresh (water/ethanol)	Dry (water)	Dry (water/ethanol)
Entire fruit*	0.84 ± 0.03	0.76 ± 0.07	3.51 ± 0.04	6.98 ± 0.01
Entire fruit**	1.07 ± 0.05	1.18 ± 0.08	5.95 ± 0.04	8.04 ± 0.02
Pulp*	0.99 ± 0.07	1.63 ± 0.17	6.74 ± 0.07	8.23 ± 0.16
Pulp**	0.97 ± 0.14	0.84 ± 0.05	7.20 ± 0.06	10.26 ± 0.04
Skin*	2.75 ± 0.02	2.93 ± 0.06	10.17 ± 0.07	14.25 ± 0.06
Skin**	2.82 ± 0.02	2.94 ± 0.03	9.18 ± 0.04	15.05 ± 0.15

*Conventional method. **MAE.

**Table 2:** Comparison of phenolic content of water extracts and 50% aqueous ethanol extracts of dry entire eggplant and skin at 100°C at variable heating times, with conventional method or microwave-assisted extraction (MAE). Values are reported in mg gallic acid equivalents ± SD (*n* = 3) on 1.5 g of eggplant.

	15 min (water)	30 min (water)	60 min (water)	90 min (water)	90 min (water/ethanol)
Entire fruit*	3.51 ± 0.04	6.66 ± 0.03	10.19 ± 0.06	14.63 ± 1.35	15.35 ± 0.19
Entire fruit**	5.95 ± 0.04	8.38 ± 0.06	21.33 ± 0.05	36.81 ± 0.04	15.19 ± 0.25
Skin*	10.17 ± 0.07	11.85 ± 0.04	21.97 ± 0.50	34.02 ± 0.16	36.13 ± 0.09
Skin**	9.18 ± 0.04	10.82 ± 0.04	18.99 ± 0.14	35.02 ± 0.16	47.09 ± 0.62

*Conventional method. **MAE.

**Table 3:** DPPH radical, superoxide anion scavenging activity, and inhibition of lipid peroxidation by different concentrations of EPS50MAE. Each value represents the mean ± SD of 4 experiments in triplicate.

	DPPH radical scavenging activity	Superoxide anion scavenging activity	Inhibition of lipid peroxidation
875 *μ*g/mL	30%	100%	40%
435 *μ*g/mL	12%	100%	20%
175 *μ*g/mL	8%	100%	10%
87.5 *μ*g/mL	0%	84%	0%
43.5 *μ*g/mL	0%	80%	0%
17.5 *μ*g/mL	0%	52%	0%
8.75 *μ*g/mL	0%	30%	0%
IC_50_ (*μ*g/mL)	>875 *μ*g/mL	17 ± 0.3 *μ*g/mL*	>875 *μ*g/mL

Values had a standard deviation of ≤10%. *P < 0.001 versus the other tests we evaluated.
